# Differences in Young Adults’ Perceptions of and Willingness to Use Nicotine Pouches by Tobacco Use Status

**DOI:** 10.3390/ijerph19052685

**Published:** 2022-02-25

**Authors:** Erin A. Vogel, Jessica L. Barrington-Trimis, Afton Kechter, Alayna P. Tackett, Fei Liu, Steve Sussman, Caryn Lerman, Jennifer B. Unger, Chanita Hughes Halbert, Benjamin W. Chaffee, Adam M. Leventhal

**Affiliations:** 1 Department of Population and Public Health Sciences, Keck School of Medicine, University of Southern California, Los Angeles, CA 90089, USA; jtrimis@usc.edu (J.L.B.-T.); kechter@usc.edu (A.K.); alayna.tackett@usc.edu (A.P.T.); feifeili@usc.edu (F.L.); ssussma@usc.edu (S.S.); unger@usc.edu (J.B.U.); hughesha@usc.edu (C.H.H.); adam.leventhal@usc.edu (A.M.L.); 2 Institute for Addiction Science, University of Southern California, Los Angeles, CA 90089, USA; caryn.lerman@med.usc.edu; 3 USC Norris Comprehensive Cancer Center, Keck School of Medicine, University of Southern California, Los Angeles, CA 90089, USA; 4 Division of Oral Epidemiology and Dental Public Health, School of Dentistry, University of California, San Francisco, CA 94118, USA; benjamin.chaffee@ucsf.edu

**Keywords:** nicotine, young adult, nicotine pouch, oral nicotine product, willingness, harm perception

## Abstract

Oral nicotine pouches may appeal to young adult current nicotine/tobacco users interested in alternative forms of nicotine that lack pulmonary exposure, but may also appeal to young adult non-users of nicotine/tobacco products. We used data from a 2020 remote digital survey of an ongoing cohort study of young adults from Southern California (aged 19–23) to examine differences in pouch perceptions and use willingness across nicotine/tobacco use statuses. Participants who had never used nicotine pouches (*N* = 1167) viewed text/imagery from mass-marketed pouch packaging and advertising, then completed measures of willingness to use nicotine pouches, pouch harm perceptions, and hypothetical choice of cigarettes or e-cigarettes over pouches. Willingness to use pouches was significantly higher among non-combustible only (33.8%), combustible only (29.3%), and dual (43.9%) users than non-users (14.7%). Overall, 49.1% of participants were uncertain whether pouches were less harmful than cigarettes and 52.4% were uncertain whether pouches were less harmful than e-cigarettes. Relative harm perceptions did not significantly differ by tobacco use status. Those using non-combustible products (either alone or as part of dual use with combustible tobacco) had greater odds than non-users of reporting that they would use e-cigarettes over nicotine pouches. By contrast, all tobacco product user groups reported greater odds than non-users that they would use cigarettes over pouches. In sum, a sizable minority of young adults might be willing to try using nicotine pouches, but most are uncertain about the relative harm of pouches.

## 1. Introduction

Nicotine pouches are a novel class of oral nicotine products, marketed as tobacco-free, in the form of pre-portioned pouches containing nicotine, flavorings, and other constituents [1]. Similar to Swedish snus, users place nicotine pouches between the lip and gum for oral nicotine absorption [1]. However, unlike snus, nicotine pouches do not contain tobacco leaves [2]. These products, including Zyn (Swedish Match), on! (Altria), Velo (R.J. Reynolds), and other brands sold by mass-market manufacturers, showed a 498% increase in unit sales at US convenience stores from early 2019 to late 2019/early 2020 [3]. In a 2019 survey of U.S. youth age 16–19, only 1.5% reported past-month nicotine pouch use; [4] however, a sizeable proportion (13%) of US young people age 15–24 reported past-month nicotine pouch use in Fall 2020 [5]. According to one major producer of nicotine pouches, U.S. shipments of nicotine pouches increased by more than 50% from 2020–2021 [6]. As nicotine pouch sales continue to increase, surveillance of use prevalence among young people will be important.

Nicotine pouches pose a regulatory dilemma when considering relative benefits and harms to nicotine/tobacco product users and non-users. Nicotine pouches may appeal to young adults who use tobacco and might be interested in alternative nicotine products that lack pulmonary exposure, but also may appeal to young adult non-users of nicotine/tobacco products. Some evidence indicates that nicotine pouches have a toxicity level lower than combustible tobacco, approaching levels comparable to nicotine replacement therapy products [7]. The relative health effects of using nicotine pouches compared to e-cigarettes and other non-combustible tobacco products (e.g., heated tobacco products, snus) are unknown [7]. However, nicotine pouches lack exposure to toxins present in some e-cigarettes, such as metals [7]. Hence, if nicotine pouches appeal to young adult users of nicotine/tobacco products and help users transition away from other nicotine/tobacco products, nicotine pouches might benefit this segment of the young adult population.

**Figure 1:**
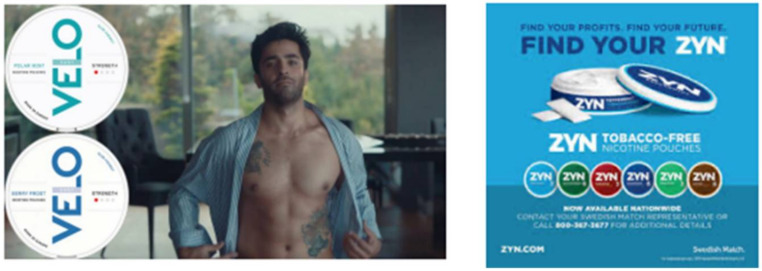
Advertisements for Velo and Zyn shown to participants.

Nicotine pouches might also appeal to young adult non-users of nicotine/tobacco products. Nicotine pouches come in a variety of flavors, such as black cherry, citrus, peppermint, and coffee [8]. Additionally, some nicotine pouch products are advertised on social media, with advertising depicting young adult models (see Figure 1). These marketing approaches have previously been used by e-cigarette manufacturers and may have increased product appeal in young non-users [9]. Nicotine pouches are easy to use and discreet relative to other tobacco products. Nicotine pouch use does not involve exhaling smoke or aerosol like inhalable tobacco products, nor does it require spitting like smokeless tobacco. Young adults who are hesitant to use inhalable products, including e-cigarettes, may nonetheless be open to trying pouches.

Because marketing of nicotine pouches is fairly new, the prevalence of nicotine pouch use might be low, making it important to understand how never-users of nicotine pouches perceive and might be willing to try these products. Whether nicotine pouch marketing and packaging differentially impact young adult nicotine/tobacco users’ and non-users’ willingness to use nicotine pouches and perception of nicotine pouches is unknown. As a first step toward assessing the impact of the increasing availability of nicotine pouches on the young adult population, it is important to understand whether young adult nicotine/tobacco users and non-users differ in their willingness to use nicotine pouches. A lack of definitive opposition to using a nicotine/tobacco product predicts greater risk of subsequent use [10,11,12]. Additionally, understanding differences between young adult nicotine/tobacco users and non-users in perceptions of the harms of nicotine pouches relative to cigarettes or e-cigarettes can help guide health messaging. Lastly, understanding choice of other products over nicotine pouches is critical to providing initial data on whether young adult nicotine/tobacco users might be interested in using nicotine pouches instead of cigarettes or e-cigarettes. The aim of this study was to compare nicotine pouch use willingness, harm perceptions, and hypothetical product choice among young adult never-users of pouches with no current nicotine/tobacco product use, exclusive non-combustible nicotine/tobacco use, exclusive combustible nicotine/tobacco use, and dual use.

## 2. Materials and Methods

### 2.1. Participants

Participants were originally recruited in 2013 from 10 high schools in the Los Angeles, California metropolitan area to participate in a longitudinal cohort study involving regular semi-annual surveys assessing health and well-being (*N* = 3396 initially enrolled in cohort) [13]. Data for the current paper used responses from a survey wave collected online May through October 2020; half of participants were randomly assigned to be administered the measures included in this study. The study was approved by the University of Southern California Institutional Review Board. Participants provided written informed consent prior to data collection.

### 2.2. Procedures

We applied the Tobacco Product Perception and Intention (TPPI) paradigm described by the US Food and Drug Administration (FDA) [14] in which participants view images of product packaging and advertising prior to perception and intention outcome assessment. Participants were first presented with a description of nicotine pouches accompanied by advertising images of the products (see Figure 1). Descriptions indicated that nicotine pouches contain no tobacco and are placed between the lip and gum, followed by the following marketing language adapted from websites from mass-marketed manufacturers of the products: “[Nicotine pouches] are advertised as a no-hands, smoke-free, spit-free and tobacco leaf-free experience. Each pouch combines nicotine salt, filler and flavoring to deliver satisfaction without smoke, spit, or odor. Some nicotine pouch brands are Zyn, Dryft, On!, and Velo. Nicotine pouches come in flavors, including cool mint, wintergreen, cinnamon, peppermint, spearmint and coffee”. Next, participants completed survey questions measuring nicotine pouch use willingness, harm perceptions, and hypothetical product choice. Participants also reported nicotine/tobacco product use and sociodemographic characteristics, described below, as part of the survey.

### 2.3. Measures

Nicotine Pouch Use Willingness, Harm Perceptions, and Hypothetical Product Choice. After viewing the marketing text and images, participants were administered five key outcome variable questions adapted from previous work for other products [10,15,16,17]. One item assessed willingness to use nicotine pouches if given the opportunity. Response options (definitely not, probably not, probably yes, and definitely yes) were recoded for analyses to indicate any willingness to using pouches (i.e., probably not/probably yes/definitely yes vs. definitely not) [10]. Two items assessed perceived harm of using nicotine pouches relative to cigarettes and to e-cigarettes (“Do you think pouches are more or less harmful than [cigarettes/e-cigarettes]?”). Both items had four response options (more harmful, about the same, less harmful, not sure). For analyses, both were recoded to indicate perceiving pouches as less harmful than cigarettes/e-cigarettes (versus more harmful, about the same, or not sure). Two items assessed hypothetical product choice, specifically, comparative likelihood of choosing to use nicotine pouches over cigarettes or e-cigarettes (“Would you be more or less likely to use pouches versus [cigarettes/e-cigarettes]?”). Response options (more likely to use nicotine pouches, equally likely, less likely to use nicotine pouches, not sure) were recoded for analyses to indicate being *less* likely to use nicotine pouches compared to cigarettes and e-cigarettes (i.e., more likely to use other products than to use pouches, equally likely to use pouches and other products, or not sure). For descriptive purposes, participants also were administered an item assessing whether they had ever heard of nicotine pouches prior to the survey (yes, no, not sure).

Past-Month Nicotine/Tobacco Product Use and Ever-Use of Nicotine Pouches. Participants reported past 30-day use (yes/no) of each of the following tobacco products: e-cigarettes (with nicotine), snus, heated tobacco products, cigarettes, cigars/cigarillos, hookah. Responses were recoded into a four-level variable reflecting past 30-day use (no use of any product, exclusive non-combustible product use [snus, e-cigarettes, or heated tobacco], exclusive combustible product use [cigarettes, cigars/cigarillos, and/or hookah], and dual use of non-combustible and combustible products). Participants also reported nicotine pouch ever-use (yes/no), which was used as a sample exclusion.

Sociodemographic Characteristics. Participants’ parental education (i.e., highest educational attainment of any parent; categorized as no high school diploma, high school diploma or some college, or college degree) was derived from the first survey wave, when participants were in their first year of high school. Family’s socioeconomic status from birth to age 16 (response options: pretty well off financially, about average, poor, it varied) was measured in the year prior to the current survey wave. At the current wave, participants reported their sex assigned at birth (male or female); race and ethnicity (categorized as non-Hispanic white, non-Hispanic Asian, non-Hispanic other race [i.e., American Indian/Alaska Native, Black, or Native Hawaiian/Pacific Islander], Hispanic white, Hispanic multiracial, or Hispanic other race); sexual identity (categorized as heterosexual or another/unreported sexual identity); and personal financial situation (response options: live comfortably, meet needs with a little left, just meet basic expenses, or don’t meet basic expenses).

### 2.4. Statistical Analysis

To characterize the overall analytic sample of nicotine pouch never-users, descriptive statistics were calculated for sociodemographic variables and each nicotine pouch use willingness and perception outcome response, including product awareness. Chi-square tests examined differences in sociodemographic characteristics by tobacco use status. Separate logistic regression models examined the association of past 30-day tobacco product use status with each of the five binary outcomes (i.e., nicotine pouch use willingness, perceived harm of nicotine pouches relative to cigarettes/e-cigarettes, and hypothetical product choice between nicotine pouches and cigarettes/e-cigarettes). Missing data on covariates were handled using a missing indicator approach; missing data on outcomes were handled using pairwise deletion. Analyses were conducted in SAS 9.4 (Cary, NC, USA) with a two-tailed 0.05 significance threshold. Benjamini–Hochberg multiple testing corrections [18] were used to control the false-discovery rate at 0.05.

## 3. Results

### 3.1. Descriptive Results in Overall Sample

Sample Characteristics. Cohort enrollees completed the Fall 2020 survey (*N* = 2437), of whom 57 (2.3%) reported previous use of nicotine pouches and were excluded from analyses. Of the remaining participants, 1207 were not randomized to receive the pouch perception measure presented in this study, and six were excluded because they did not provide data on their current tobacco product use. The analytic sample (*N* = 1167; see Table 1 for demographic characteristics) was 60.1% female (39.9% male), 54.2% Hispanic (19.2% non-Hispanic Asian, 11.8% non-Hispanic white, and 30.9% non-Hispanic other race), and 21.3% reported a sexual identity other than heterosexual. Approximately half of participants (49.8%) described their family’s financial status as about average and 53.6% had a parent with a college degree. A plurality (43.5%) reported they live comfortably; 31.1% met their needs with a little left, 22.0% just met basic expenses, and 3.4% did not meet basic expenses. Regarding current tobacco product use status, 916 (78.5%) reported no use of any tobacco products, 140 (12.0%) exclusive use of e-cigarettes or other non-combustible tobacco products, 42 (3.6%) exclusive use of combustible tobacco products, and 69 (5.9%) dual use of combustible and non-combustible tobacco products. Sex, race/ethnicity, and sexual identity were significantly associated with tobacco use status (*p*-values < 0.05).

Nicotine Pouch Use Willingness and Perceptions. The frequencies of each response option (before collapsing categories for the primary analysis) for each outcome are reported in Table 2. Most participants (82.4%) reported having never heard of nicotine pouches before taking this survey (Table 2). Although 19.1% of the overall sample reported being willing to using nicotine pouches (i.e., were not definitely opposed to using them), only 0.7% said they would definitely use them. Nearly half (49.1%) of participants were unsure whether nicotine pouches were more or less harmful than smoking cigarettes. Similar proportions viewed nicotine pouches as less harmful (19.7%) or about the same harm (20.9%) as cigarettes, with 10.3% considering nicotine pouches to be more harmful. Similarly, 52.4% of participants were unsure how the harm of nicotine pouches compared to e-cigarettes; 13.6% viewed nicotine pouches as less harmful, 12.2% as more harmful, and 21.7% about the same. When asked about whether they would choose nicotine pouches over combustible cigarettes, an appreciable portion of the sample was uncertain (57.3%); 23.2% reported lower likelihood of using nicotine pouches than cigarettes, 10.4% reported equal likelihood of using cigarettes and nicotine pouches, and 9.1% reported greater likelihood of using nicotine pouches than cigarettes. Relative likelihood of nicotine pouch use compared to e-cigarettes showed a similar pattern, with 29.7% reporting lower likelihood of using nicotine pouches compared to e-cigarettes.

### 3.2. Association of Tobacco Product Use Status with Nicotine Pouch Use Willingness and Perceptions

Compared to non-users, young adults using combustible and/or non-combustible tobacco products were significantly more likely to be willing to use nicotine pouches (among non-users: 14.7%, exclusive non-combustible product users: 33.8%, exclusive combustible product users: 29.3%, dual users: 43.9%; ORs = 2.29–4.27, *ps* < 0.024) (Table 3). Tobacco product use status was not associated with perception of nicotine pouches as less harmful than cigarettes (among non-users: 18.8%, non-combustible product users: 24.8%, combustible product users: 9.8%, dual users: 27.3%; ORs = 0.45–1.46; *ps* > 0.138) or less harmful than e-cigarettes (non-users: 13.6%, non-combustible product users: 15.3%, combustible product users: 4.9%, dual users: 16.9%; ORs = 0.30–1.19; *ps* > 0.104). Hypothetical choice of other e-cigarettes over nicotine pouches was concordant with participants’ tobacco use status, such that those using e-cigarettes or other non-combustible products (either alone or as part of dual use with combustible tobacco) had greater odds than non-users of reporting that they would use e-cigarettes over nicotine pouches, but exclusive combustible product users and tobacco non-users did not differ in this outcome (Table 3). By contrast, all tobacco product use groups reported greater odds than non-users that they would choose cigarettes over pouches (among non-users: 17.7%, non-combustible product users: 39.4%, combustible product users: 40.0%, dual users: 55.4%; ORs = 3.19–5.76; *ps* < 0.002).

## 4. Discussion

This study provides new evidence regarding the potential implications of nicotine pouches for young adult nicotine users and non-users. We found that after being shown advertising materials for nicotine pouch products, a sizable minority of young adults—mostly those currently using combustible and/or non-combustible tobacco—were willing to use nicotine pouches if given the opportunity. Most young adults did not perceive the harm of nicotine pouches as being greater or less than cigarettes or e-cigarettes, and many were uncertain about the relative harms. Young adults currently using tobacco products were generally more likely than non-users to choose cigarettes and e-cigarettes over pouches.

A previous analysis of consumer data collected from November 2017 to February 2018 by Swedish Match, the manufacturer of Zyn, suggested that Zyn appealed to adult current users of cigarettes and smokeless oral tobacco, with low appeal to non-users [1]. The current data are consistent with the previous analysis of Zyn manufacturer-collected data in a general adult consumer panel sample, in the sense that willingness to use nicotine pouches in this study was substantially more common among tobacco product users than non-users, regardless of whether young adults were exclusively using combustible products, exclusively using non-combustible products, or dual using [18]. However, the non-negligible prevalence of willingness to use nicotine pouches among tobacco non-users (14.7%) suggests that a large number of young adults could initiate nicotine use with pouches. Nicotine pouch use could be beneficial to young adults currently using tobacco if they switch entirely from inhalable tobacco products (e.g., cigarettes and e-cigarettes) to nicotine pouches. However, uptake of nicotine pouch use could harm tobacco non-users by exposing them to nicotine. Prevalence of nicotine pouch initiation among young adults, both tobacco users and non-users, is warranted to understand the impact of nicotine pouch sales on population health. Measures assessing participants’ comparative likelihood of choosing to use nicotine pouches over other products revealed that in the overall sample, cigarettes and e-cigarettes were more appealing than nicotine pouches. Hence, there may be a low overall likelihood that young adults who use tobacco products would consider switching to using nicotine pouches merely after viewing product packaging and marketing.

Large proportions of young adults in this study were unsure whether nicotine pouches were more or less harmful than cigarettes (49.1%) and e-cigarettes (52.4%). While more data need to be collected about the health effects of nicotine pouches, initial toxicology data and biological plausibility provide a strong premise that nicotine pouches are likely to be far less harmful than combustible tobacco [5], and they lack pulmonary exposures present in all inhalable tobacco products, including e-cigarettes. Our findings indicate that the average young adult user, regardless of their tobacco product use status, is likely to be unaware of the important possible differences between nicotine pouches and other products. Similarly, most U.S. adults believe e-cigarettes and smokeless tobacco are at least as harmful as cigarettes or are unsure about relative harms [19]. Current messaging around nicotine/tobacco products may not fully explain relative harms.

Snus moist snuff oral tobacco products manufactured by Swedish Match have been authorized by the U.S. Food and Drug Administration (FDA) as modified risk tobacco products (MRTPs) that can be legally marketed with claims of reduced harm relative to combustible cigarettes [20]. The nicotine delivery and possible abuse liability of snus and nicotine pouches appear to be similar [21]. Nicotine pouches may contain less tobacco leaf material than snus, given they are marketed as ‘tobacco free,’ and may contain fewer toxins than snus. For these reasons, nicotine pouch manufacturers could potentially pursue an MRTP designation in the future. Our findings suggest that MRTP claims could address a lack of knowledge from the general young adult population about the relative harms of pouches compared to cigarettes and other inhalable products. Future research should examine whether modified risk marketing claims accompanying nicotine pouches change harm perception and use willingness for both users and non-users of tobacco products. Such data would be critical to guide FDA if a nicotine pouch manufacturer submits an MRTP application and if nicotine pouch sales continue to increase.

Although not the focus of the study, it is worth noting that only 2.3% of young adults in this sample surveyed in 2020 had ever used nicotine pouches and 10.6% of those who had never used nicotine pouches reported being aware of them. Low product awareness is consistent with data from a 2019 online survey of UK adults who currently or formerly smoked or vaped. Only 15.9% of surveyed adults were aware of nicotine pouches, despite their current or former tobacco product use [22]. Moreover, a large majority of participants in this study (80.9%) would “definitely not” use nicotine pouches. This finding is consistent with data from U.S. youth surveyed in 2019, of whom only 1.5% reported past-month nicotine pouch use [4]. However, product awareness and use among young people may increase over time. Nielsen data show a large increase in nicotine pouch sales in recent years, from $709,635 in 2016 to $216,886,819 in the first half of 2020. Fruit-flavored nicotine pouches showed the largest increase in unit sales from January 2019–June 2020, compared to other flavors [23]. It is possible that as awareness grows, perceptions of nicotine pouches may also shift and solidify.

### Limitations and Future Directions

Results should be interpreted in light of a few limitations. First, all participants were recruited from a school-based cohort study in the Los Angeles, California metropolitan area. Results may not generalize across geographic areas or to young adults who left high school prior to graduation or were lost to attrition after high school. Second, the relatively small number of combustible product users may have limited statistical power to detect differences in use willingness and perceptions by tobacco use status and to examine sociodemographic characteristics as potential moderators. Third, survey items measured harm perceptions relative to cigarettes and e-cigarettes only. Future research could examine differences in absolute harm perceptions by tobacco use status. Furthermore, measures assessing hypothetical product choice did not assess actual product-switching behavior, which merits examination. Participants are enrolled in an ongoing longitudinal cohort study, enabling examination of prospective associations of willingness to use nicotine pouches with reported product use. Fourth, participants were exposed only briefly to nicotine pouch marketing materials, and baseline harm perceptions prior to advertising exposure were not measured. Repeated exposure may have a stronger impact on use willingness and perceptions. Future research could examine a dose–response relationship between advertising exposure, willingness, and perceptions.

## 5. Conclusions and Implications

Uncertainty about the harms of nicotine pouches was common in this sample of young adults, and willingness to use nicotine pouches may be disproportionately prevalent among (but not limited to) young adults who use tobacco products. Consequently, despite low nicotine pouch use prevalence currently, it is possible that increasing marketing and sales of nicotine pouches in the future could ultimately impact young adult health either positively or negatively. Whether such impact is driven by young adult tobacco users who switch to nicotine pouch use or by non-users drawn into nicotine/tobacco product use via nicotine pouches remains to be seen. Further investigation is warranted to examine the relative harms of using nicotine pouches versus other products and whether nicotine pouch marketing selectively attracts young adults who use tobacco.

## Figures and Tables

**Table 1:** Participant characteristics (*N* = 1167) by past 30-day tobacco product use status.

Variable		No Use of Any Tobacco Product (*n* = 916)	Non-Combustible Only (*n* = 140)	Combustible Only (*n* = 42)	Dual Use (*n* = 69)	Overall Sample (*N* = 1167)
		*n* (%)				
Sex assigned at birth *	Male	337 (37.5)	61 (45.9)	20 (51.3)	36 (53.7)	454 (39.9)
	Female	562 (62.5)	72 (54.1)	19 (48.7)	31 (46.3)	684 (60.1)
Race/ethnicity *	Non-Hispanic White	98 (10.9)	13 (9.8)	6 (15.8)	17 (25.4)	134 (11.8)
	Hispanic White	107 (11.9)	13 (9.8)	8 (21.1)	4 (6.0)	132 (11.6)
	Hispanic multi-racial	99 (11.0)	12 (9.1)	7 (18.4)	14 (20.9)	132 (11.6)
	Hispanic Other	287 (32.0)	40 (30.3)	8 (21.1)	16 (23.9)	351 (30.9)
	Non-Hispanic Asian	176 (19.6)	31 (23.5)	4 (10.5)	7 (10.4)	218 (19.2)
	Non-Hispanic Other	131 (14.6)	23 (17.4)	5 (13.2)	9 (13.4)	168 (14.8)
Sexual identity *	Heterosexual	719 (80.2)	108 (81.2)	25 (65.8)	40 (59.7)	892 (78.7)
	Another or unreported sexual identity ^a^	177 (19.8)	25 (18.8)	13 (34.2)	27 (40.3)	242 (21.3)
Socioeconomic status (family) ^b^	Pretty well off financially	193 (23.3)	21 (16.8)	9 (24.3)	13 (22.8)	236 (22.5)
	About average	416 (50.2)	60 (48.0)	18 (48.6)	28 (49.1)	522 (49.8)
	Poor	130 (15.7)	27 (21.6)	5 (13.5)	12 (21.1)	174 (16.6)
	It varied	90 (10.9)	17 (13.6)	5 (13.5)	4 (7.0)	116 (11.1)
Socioeconomic status (self) ^c^	Live comfortably	398 (44.5)	53 (39.8)	14 (36.8)	27 (40.9)	492 (43.5)
	Meet needs with a little left	284 (31.7)	41 (30.8)	11 (28.9)	16 (24.2)	352 (31.1)
	Just meet basic expenses	184 (20.6)	36 (27.1)	12 (31.6)	17 (25.8)	249 (22.0)
	Don’t meet basic expenses	29 (3.2)	3 (2.3)	1 (2.6)	6 (9.1)	39 (3.4)
Parental education (youth)	No high school diploma	88 (11.0)	17 (13.2)	7 (18.4)	4 (6.5)	116 (11.3)
	High school diploma or some college	278 (34.8)	44 (34.1)	13 (34.2)	26 (41.9)	361 (35.1)
	College degree	433 (54.2)	68 (52.7)	18 (47.4)	32 (51.6)	551 (53.6)
Age	Less than 21 years old	339 (37.0)	44 (31.4)	13 (31.0)	20 (29.0)	416 (35.7)
	21 years or older	576 (63.0)	96 (68.6)	29 (69.0)	49 (71.0)	750 (64.3)

Note: Analytic sample *N* = 1167. Percentages reflect proportion of participants with non-missing data on each characteristic. ^a^ Includes asexual, bisexual, gay, lesbian, pansexual, queer, questioning or unsure, another identity, or “prefer not to disclose”. ^b^ Perceived socioeconomic status of one’s family from birth to age 16. ^c^ Perceived current socioeconomic status considering the participant’s own income and other financial support received. * Significantly associated with tobacco use status in chi-square tests (*p* < 0.05).

**Table 2:** Nicotine pouch use willingness and perceptions by tobacco product use status.

Variable (*n*/%)	No Use of Any Tobacco Product	Non-Combustible Only	Combustible Only	Dual Use	Full Sample
Aware of nicotine pouches before survey					
Yes	79 (8.8)	25 (18.7)	4 (9.8)	13 (19.7)	121 (10.6)
No	757 (84.1)	101 (75.4)	33 (80.5)	49 (74.2)	940 (82.4)
Not sure	64 (7.1)	8 (6.0)	4 (9.8)	4 (6.1)	80 (7.0)
Willingness to use nicotine pouches if given the opportunity					
Definitely Not	763 (85.3)	88 (66.2)	29 (70.7)	37 (56.1)	917 (80.9)
Probably Not	112 (12.5)	34 (25.6)	11 (26.8)	16 (24.2)	173 (15.3)
Probably Yes	14 (1.6)	10 (7.5)	1 (2.4)	11 (16.7)	36 (3.2)
Definitely Yes	5 (0.6)	1 (0.8)	0 (0)	2 (3.0)	8 (0.7)
Nicotine pouch harm perceptions relative to cigarettes					
Nicotine pouches more harmful	84 (9.4)	18 (13.5)	6 (14.6)	9 (13.6)	117 (10.3)
About the same	186 (20.7)	29 (21.8)	8 (19.5)	15 (22.7)	238 (20.9)
Nicotine pouches less harmful	169 (18.8)	33 (24.8)	4 (9.8)	18 (27.3)	224 (19.7)
Not sure	459 (51.1)	53 (39.8)	23 (56.1)	24 (36.4)	559 (49.1)
Nicotine pouch harm perceptions relative to e-cigarettes					
Nicotine pouches more harmful	99 (11.0)	21 (16.0)	8 (19.5)	11 (16.9)	139 (12.2)
About the same	194 (21.6)	32 (24.4)	9 (22.0)	12 (18.5)	247 (21.7)
Nicotine pouches less harmful	122 (13.6)	20 (15.3)	2 (4.9)	11 (16.9)	155 (13.6)
Not sure	484 (53.8)	58 (44.3)	22 (53.7)	31 (47.7)	595 (52.4)
Likely to use nicotine pouches versus smoking cigarettes					
More likely to use nicotine pouches vs. cigarettes	81 (9.1)	14 (10.6)	3 (7.5)	5 (7.7)	103 (9.1)
Equally likely	103 (11.5)	8 (6.1)	3 (7.5)	3 (4.6)	117 (10.4)
Less likely to use nicotine pouches vs. cigarettes	158 (17.7)	52 (39.4)	16 (40.0)	36 (55.4)	262 (23.2)
Not sure	551 (61.7)	58 (43.9)	18 (45.0)	21 (32.3)	648 (57.3)
Likely to use nicotine pouches versus using e-cigarettes					
More likely to use nicotine pouches vs. e-cigarettes	52 (5.8)	2 (1.5)	2 (4.9)	5 (7.7)	61 (5.4)
Equally likely	95 (10.6)	5 (3.8)	3 (7.3)	3 (4.6)	106 (9.4)
Less likely to use nicotine pouches vs. e-cigarettes	214 (23.9)	72 (54.5)	14 (34.1)	37 (56.9)	337 (29.7)
Not sure	534 (59.7)	53 (40.2)	22 (53.7)	20 (30.8)	629 (55.5)

**Table 3:** Associations of past 30-day tobacco use status with nicotine pouch use willingness and perceptions.

Outcome	Past 30-Day Tobacco Product Use Status			
	No Use of Any Tobacco Product	Non-Combustible Only	Combustible Only	Dual Use
Willing to use nicotine pouches if had opportunity				
*n* (%) willing	131 (14.7)	45 (33.8)	12 (29.3)	29 (43.9)
OR (95% CI)	Ref.	2.99 (1.99, 4.49) *	2.29 (1.12, 4.68) *	4.27 (2.49, 7.32) *
Perceive nicotine pouches as less harmful than smoking cigarettes				
*n* (%) perceive less harm	169 (18.8)	33 (24.8)	4 (9.8)	18 (27.3)
OR (95% CI)	Ref.	1.36 (0.88, 2.11)	0.45 (0.16, 1.29)	1.46 (0.81, 2.64)
Perceive nicotine pouches as less harmful than using e-cigarettes				
*n* (%) perceive less harm	122 (13.6)	20 (15.3)	2 (4.9)	11 (16.9)
OR (95% CI)	Ref.	1.15 (0.68, 1.94)	0.30 (0.07, 1.29)	1.19 (0.59, 2.40)
More likely to smoke cigarettes than use nicotine pouches				
*n* (%) more likely	158 (17.7)	52 (39.4)	16 (40.0)	36 (55.4)
OR (95% CI)	Ref.	3.28 (2.21, 4.88) *	3.19 (1.62, 6.27) *	5.76 (3.36, 9.88) *
More likely to use e-cigarettes than use nicotine pouches				
*n* (%) more likely	214 (23.9)	72 (54.5)	14 (34.1)	37 (56.9)
OR (95% CI)	Ref.	4.18 (2.85, 6.14) *	1.53 (0.77, 3.04)	4.06 (2.38, 6.92) *

Note: Logistic regression analyses adjusted for sex, race/ethnicity, and sexual identity. * *p* < 0.05 after correction for multiple testing.

## Data Availability

The data presented in this study are available on request from the corresponding author. The data are not publicly available because the longitudinal cohort study from which data were drawn is ongoing.
